# Supplementary figures and images for: APOE expression and secretion are modulated by mitochondrial dysfunction
Source: eLife. 2023 May 12;12:e85779. doi: 10.7554/eLife.85779 (PMC10231934; doi:10.7554/eLife.85779)

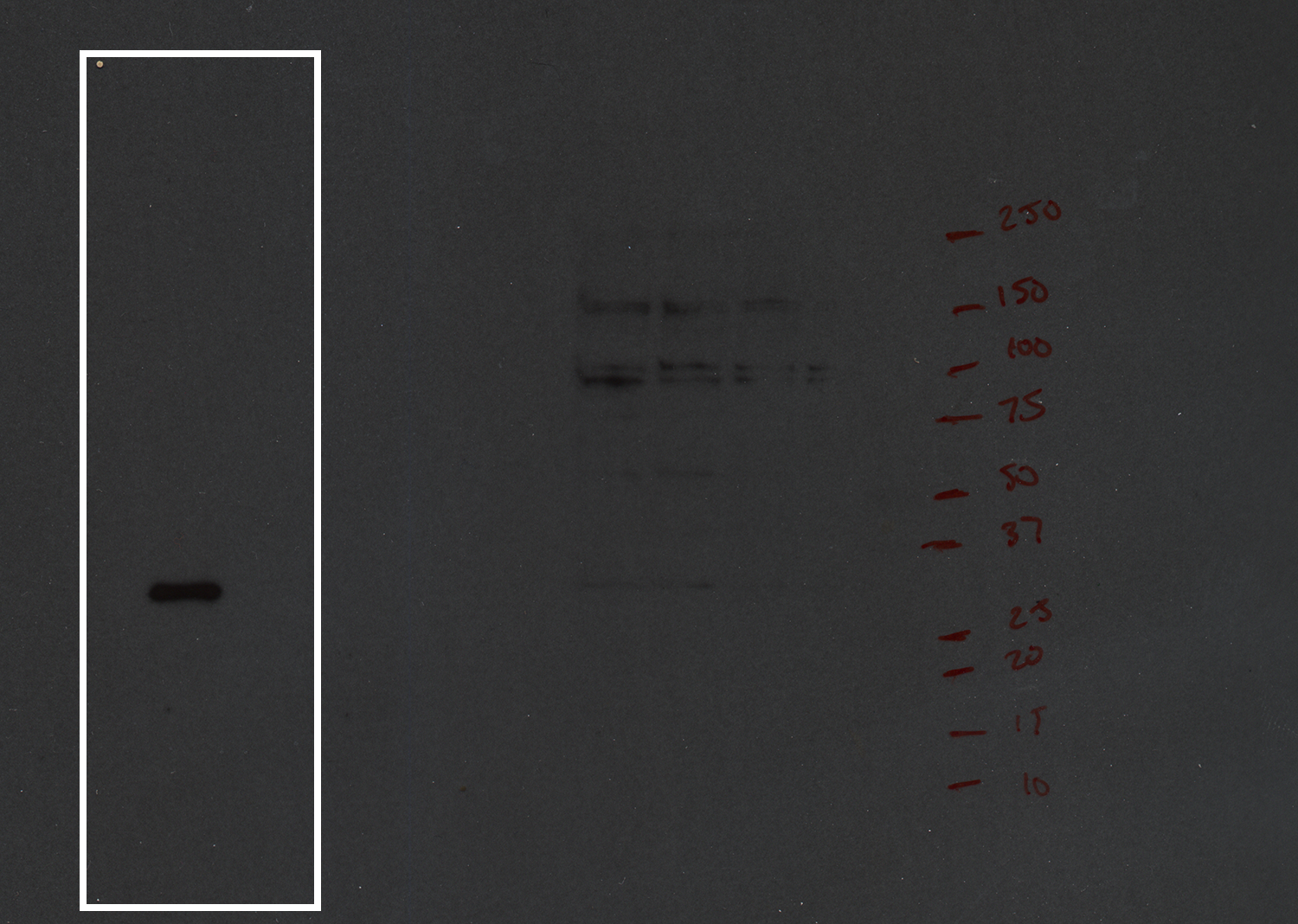

Supplement: Figure 2—source data 1. [file elife-85779-fig2-data1.zip › Figure 2B Source Data/Figure 2B Source Data 4.tif]

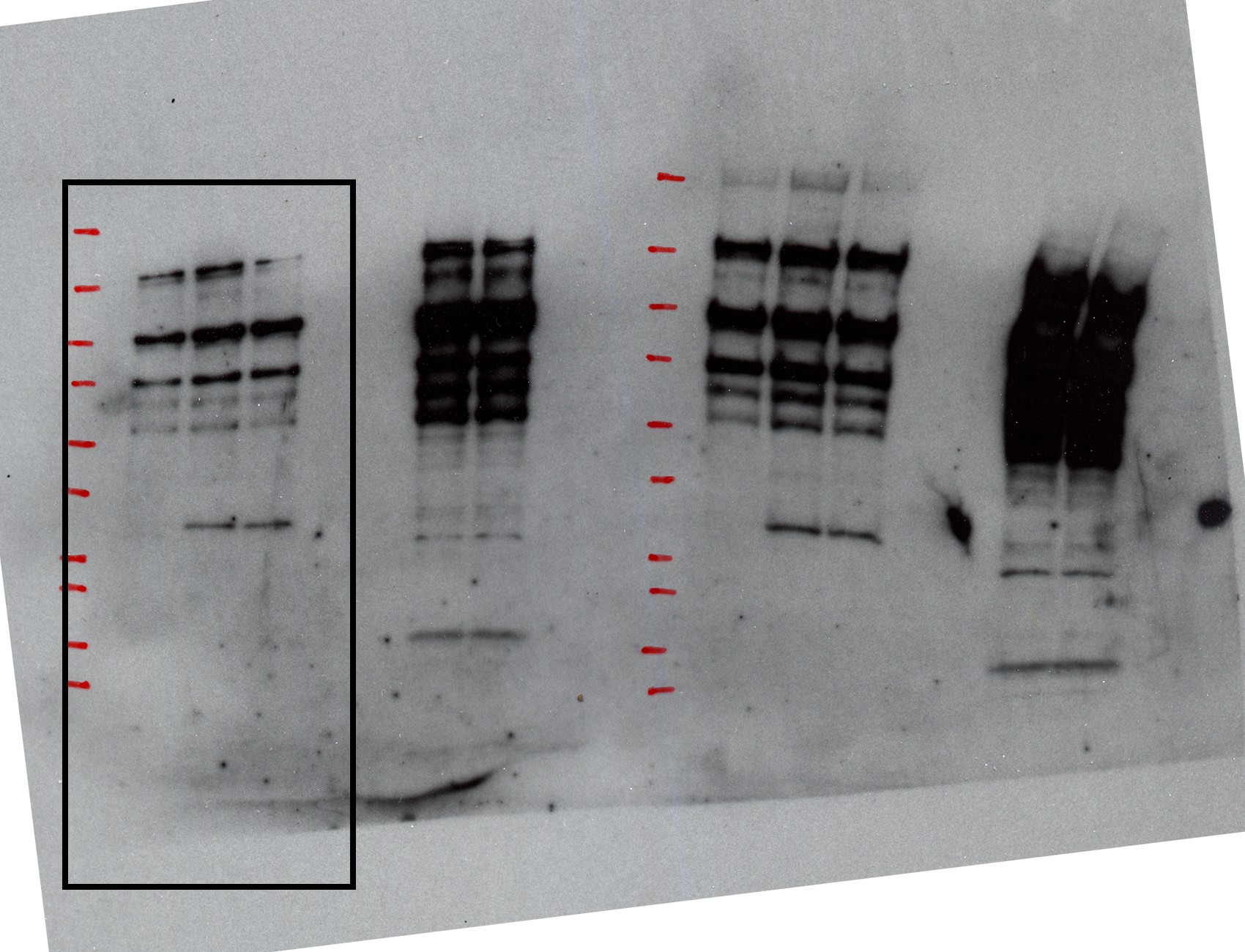

Supplement: Figure 2—source data 1. [file elife-85779-fig2-data1.zip › Figure 2B Source Data/Figure 2B Source Data 1.tif]

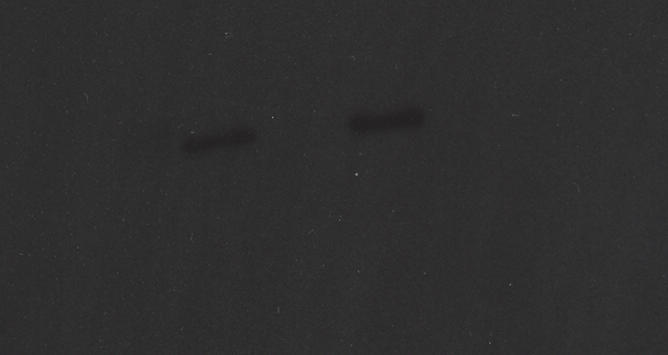

Supplement: Figure 2—source data 1. [file elife-85779-fig2-data1.zip › Figure 2B Source Data/Figure 2B Source Data 3.tif]

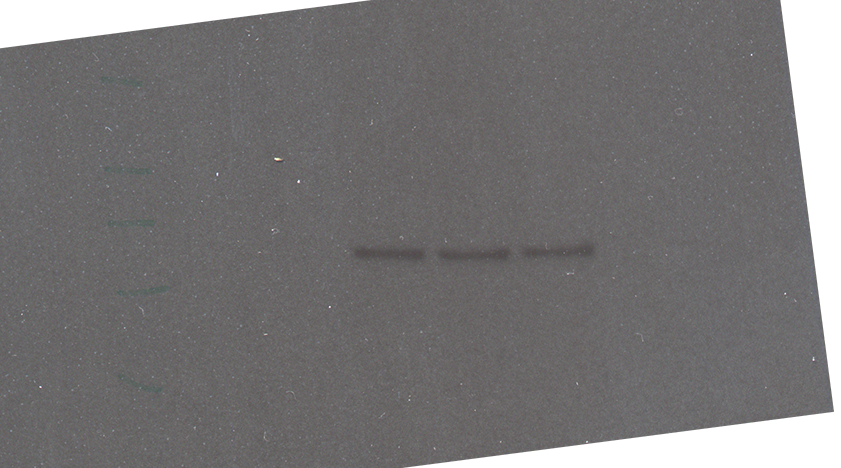

Supplement: Figure 2—source data 1. [file elife-85779-fig2-data1.zip › Figure 2B Source Data/Figure 2B Source Data 2.tif]

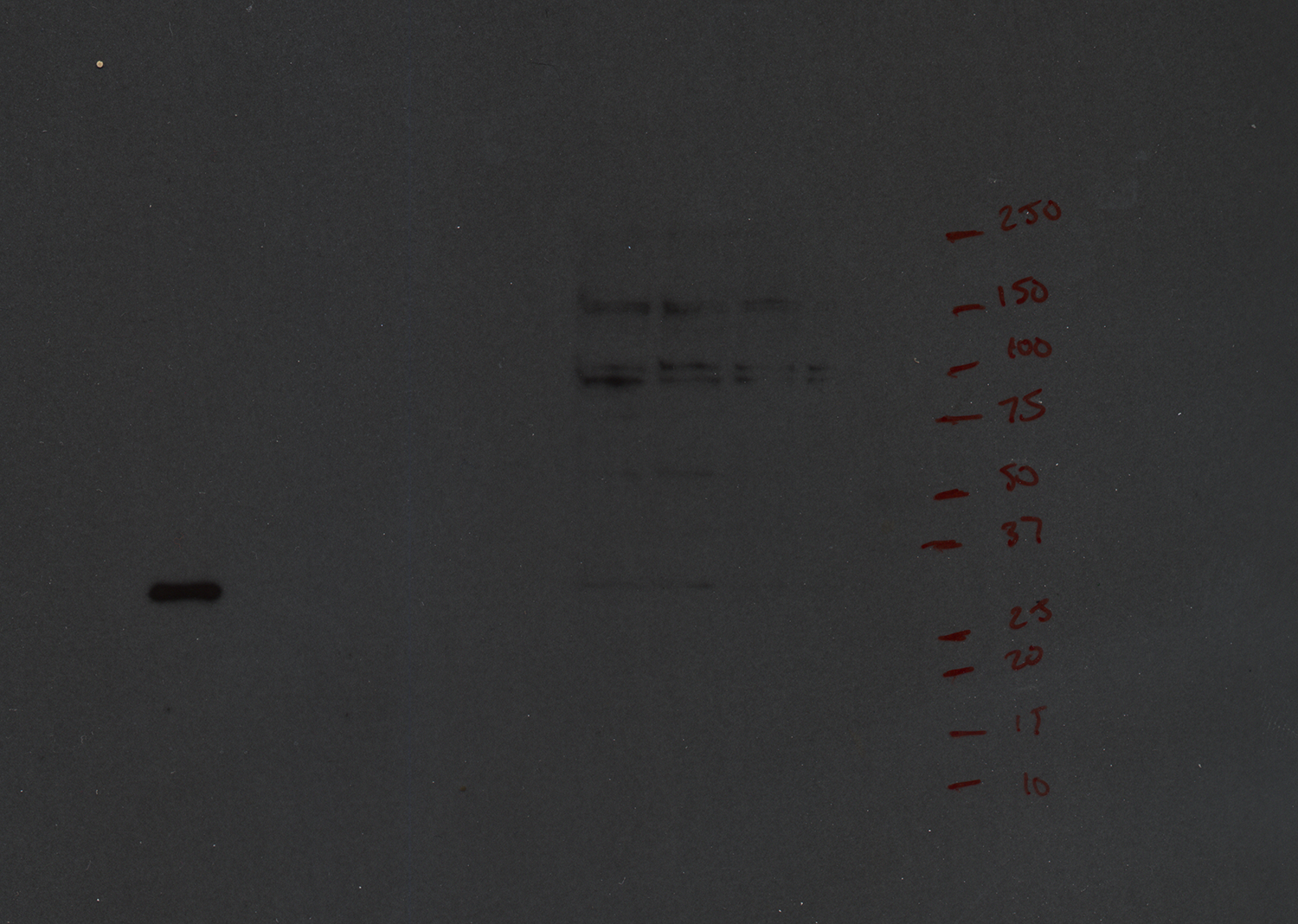

Supplement: Figure 2—source data 2. [file elife-85779-fig2-data2.zip › Figure 2B Source Data Unmarked/Figure 2B Source Data 4.tif]

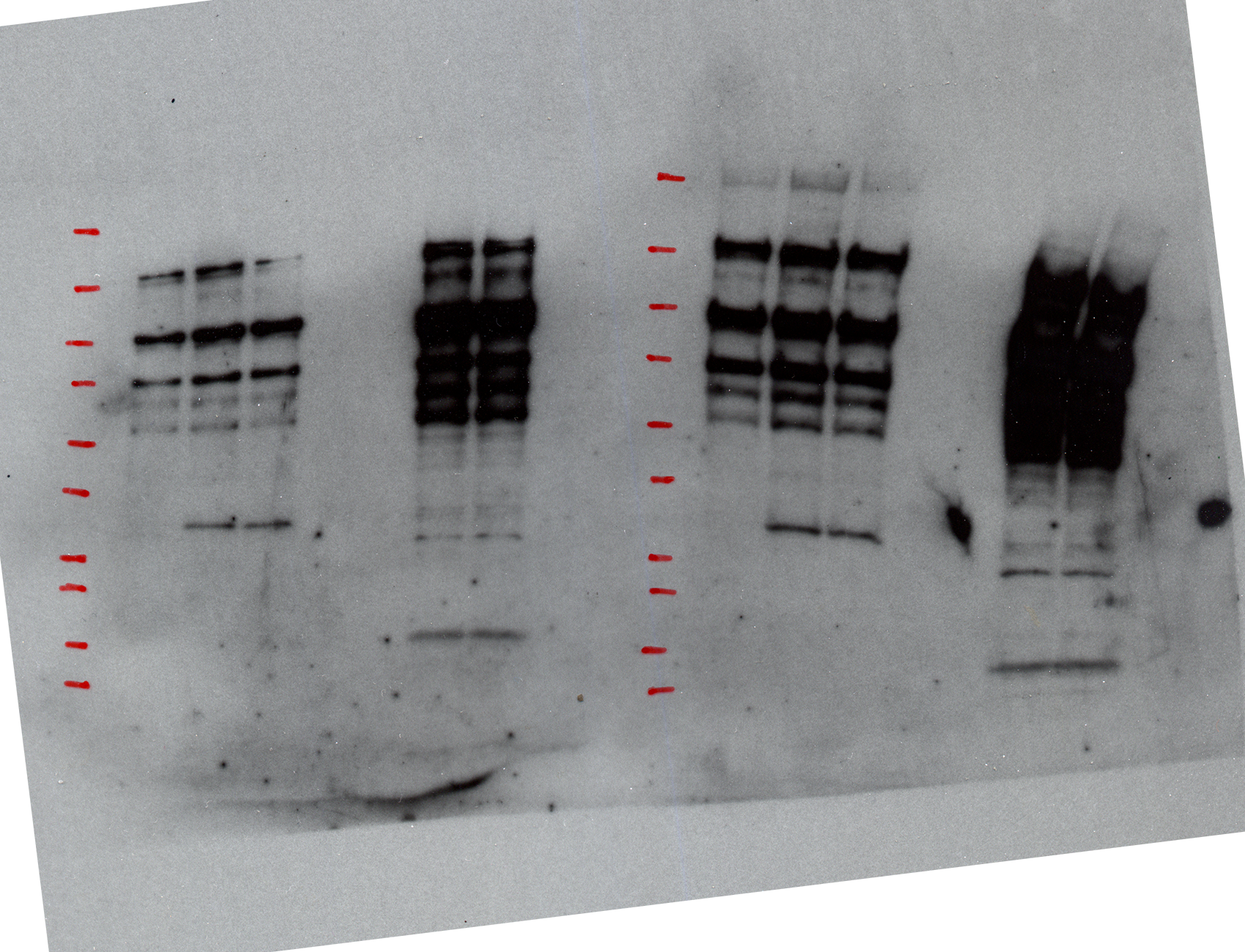

Supplement: Figure 2—source data 2. [file elife-85779-fig2-data2.zip › Figure 2B Source Data Unmarked/Figure 2B Source Data 1.tif]

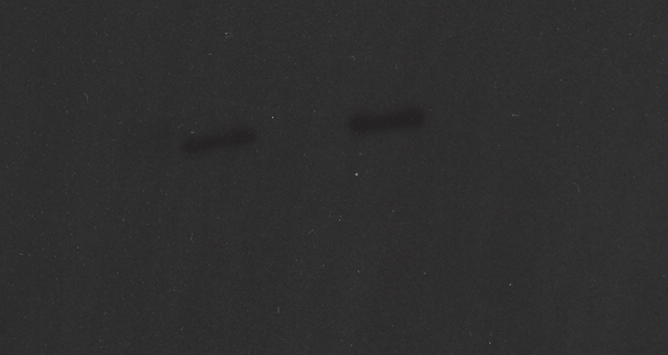

Supplement: Figure 2—source data 2. [file elife-85779-fig2-data2.zip › Figure 2B Source Data Unmarked/Figure 2B Source Data 3.tif]

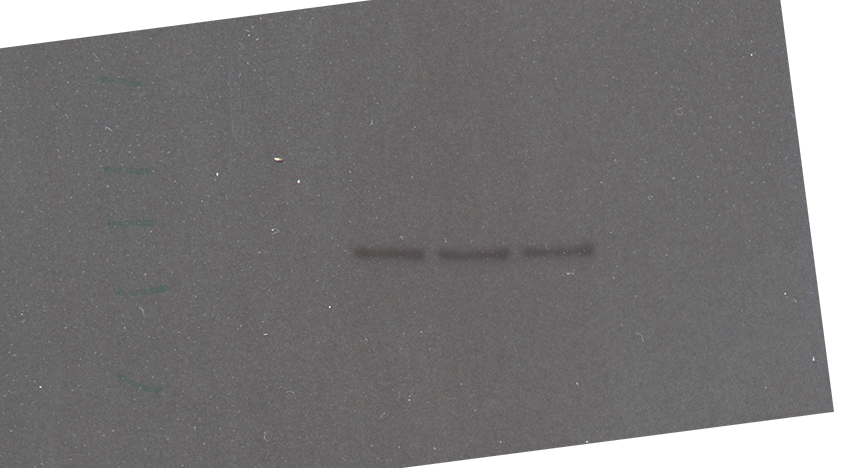

Supplement: Figure 2—source data 2. [file elife-85779-fig2-data2.zip › Figure 2B Source Data Unmarked/Figure 2B Source Data 2.tif]

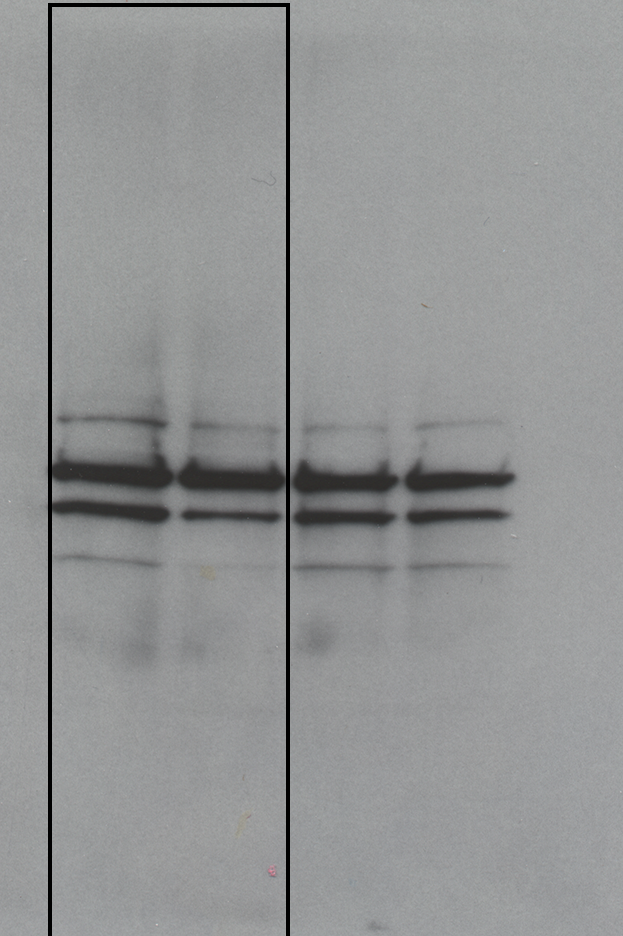

Supplement: Figure 3—source data 1. [file elife-85779-fig3-data1.zip › Figure 3 Source Data/Figure 3B Source Data 1.tif]

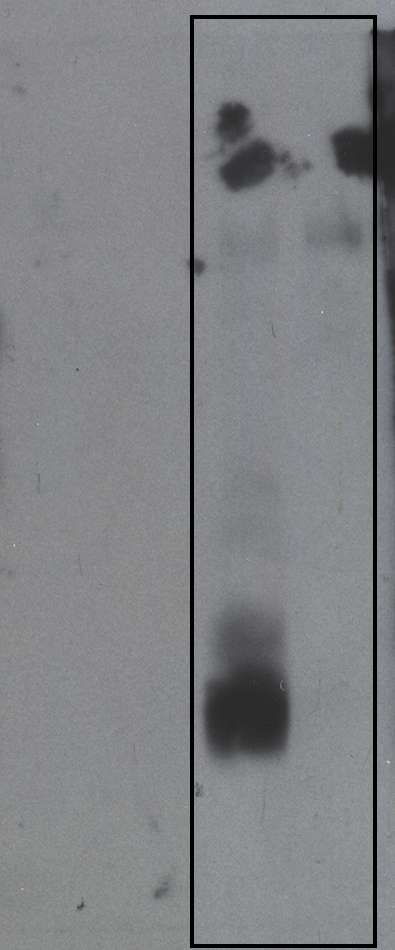

Supplement: Figure 3—source data 1. [file elife-85779-fig3-data1.zip › Figure 3 Source Data/Figure 3C Source Data 4.tif]

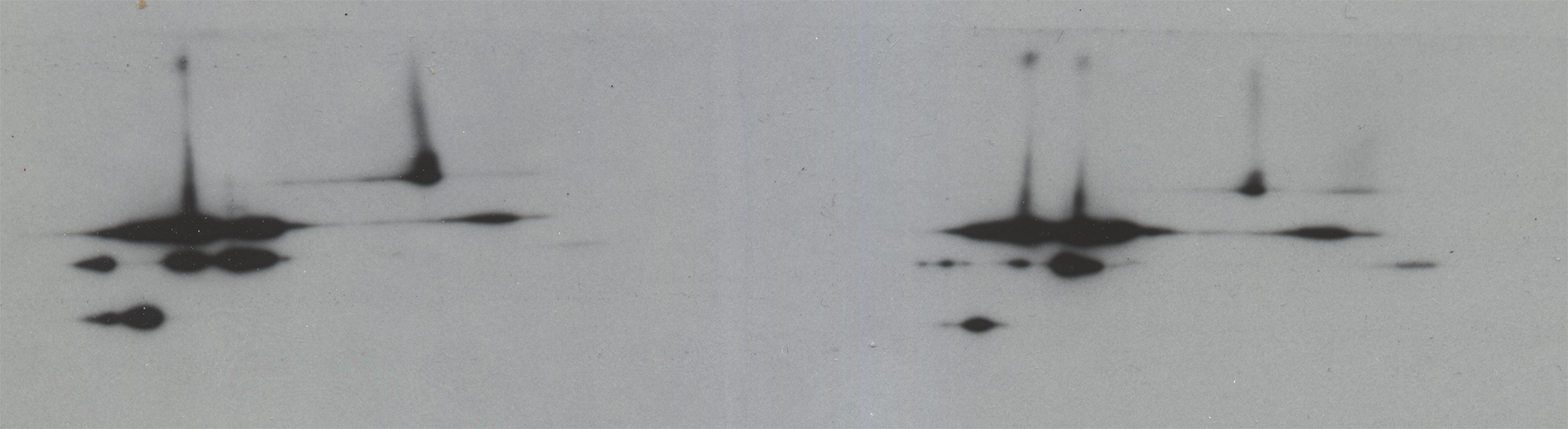

Supplement: Figure 3—source data 1. [file elife-85779-fig3-data1.zip › Figure 3 Source Data/Figure 3C Source Data 5.tif]

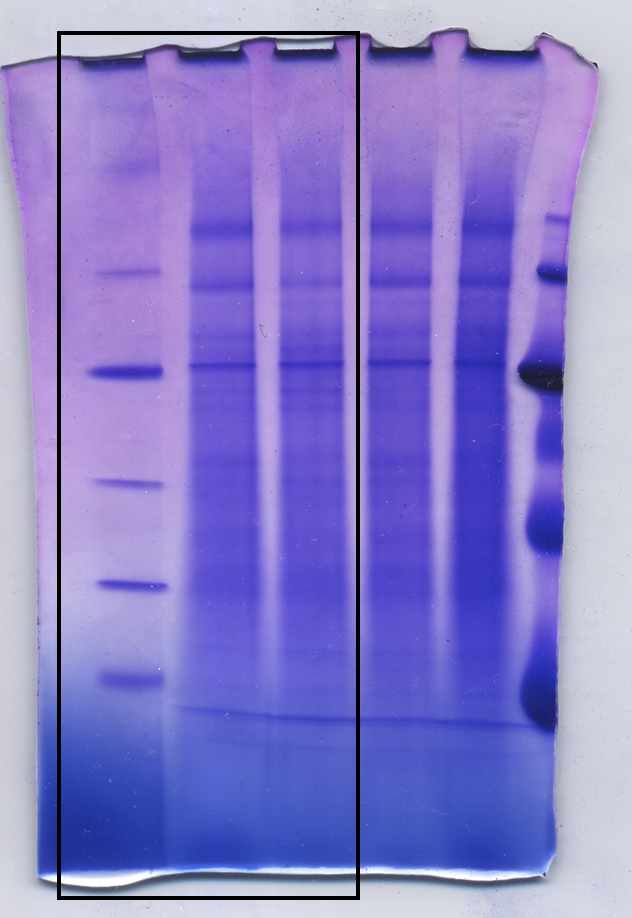

Supplement: Figure 3—source data 1. [file elife-85779-fig3-data1.zip › Figure 3 Source Data/Figure 3C Source Data 1.tif]

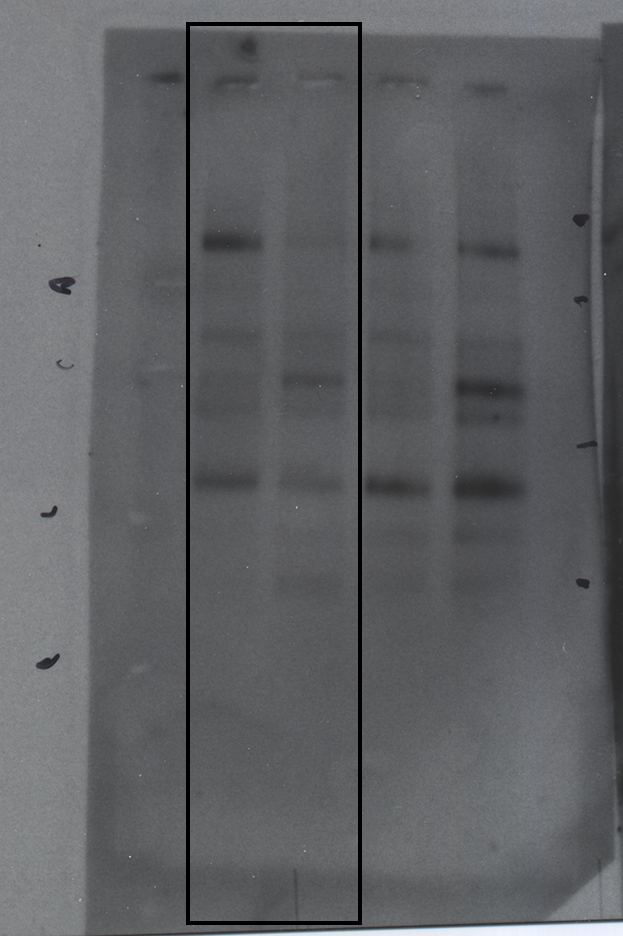

Supplement: Figure 3—source data 1. [file elife-85779-fig3-data1.zip › Figure 3 Source Data/Figure 3C Source Data 2.tif]

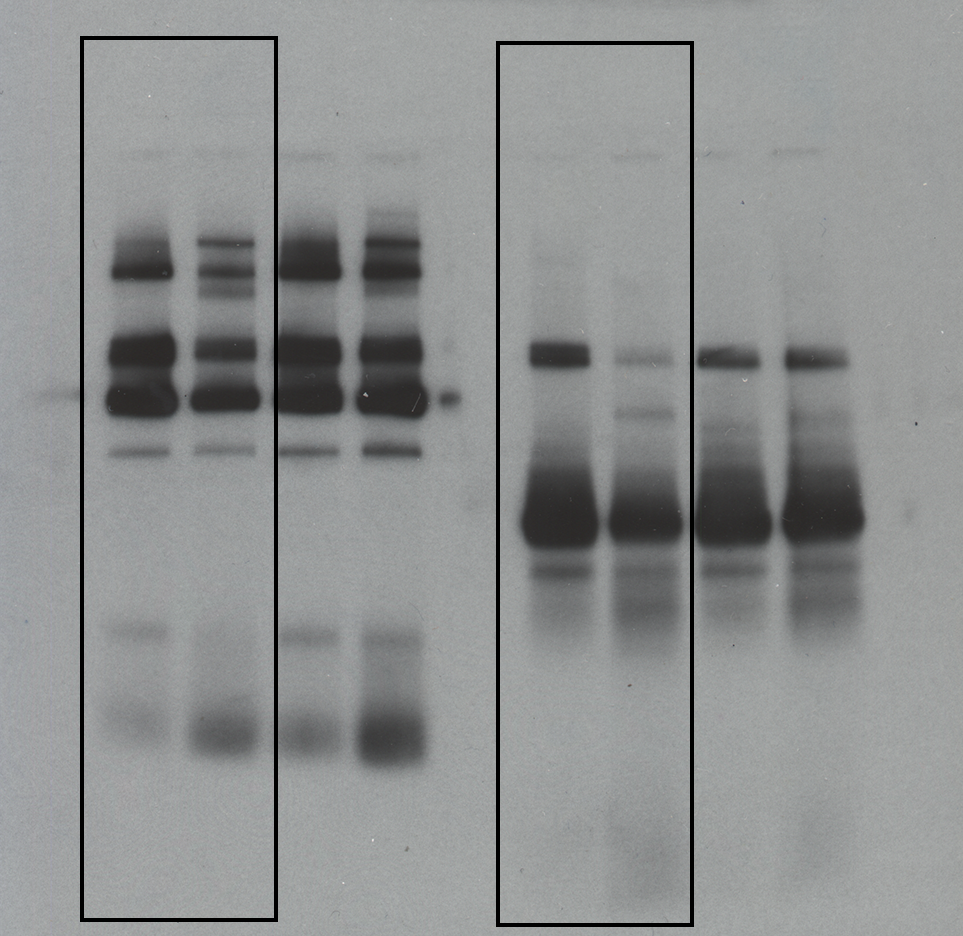

Supplement: Figure 3—source data 1. [file elife-85779-fig3-data1.zip › Figure 3 Source Data/Figure 3C Source Data 3.tif]

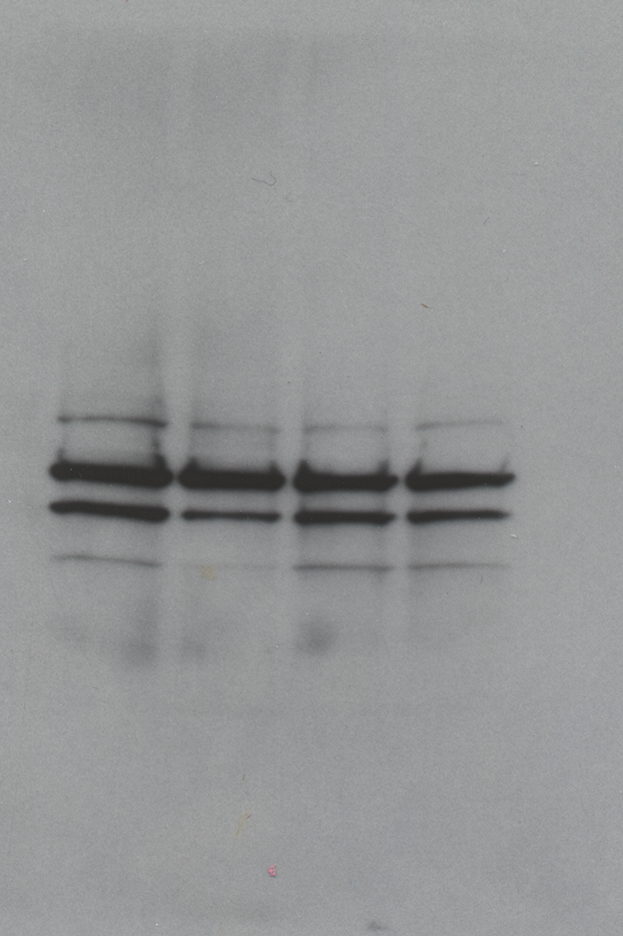

Supplement: Figure 3—source data 2. [file elife-85779-fig3-data2.zip › Figure 3 Source Data Unmarked/Figure 3B Source Data 1.tif]

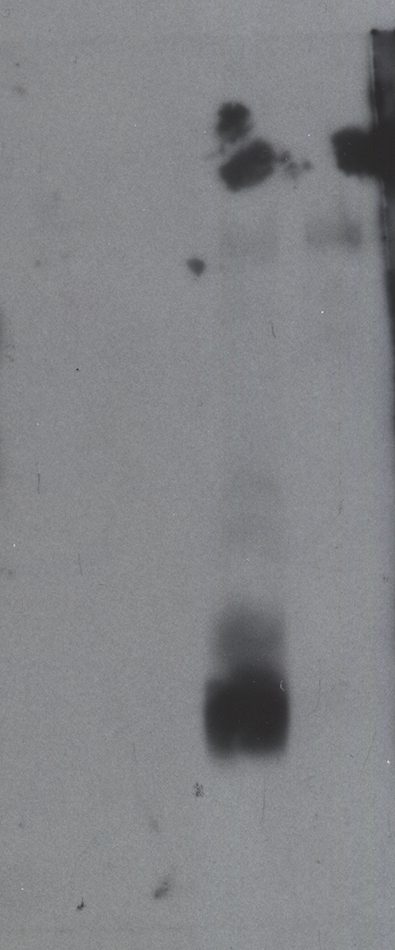

Supplement: Figure 3—source data 2. [file elife-85779-fig3-data2.zip › Figure 3 Source Data Unmarked/Figure 3C Source Data 4.tif]

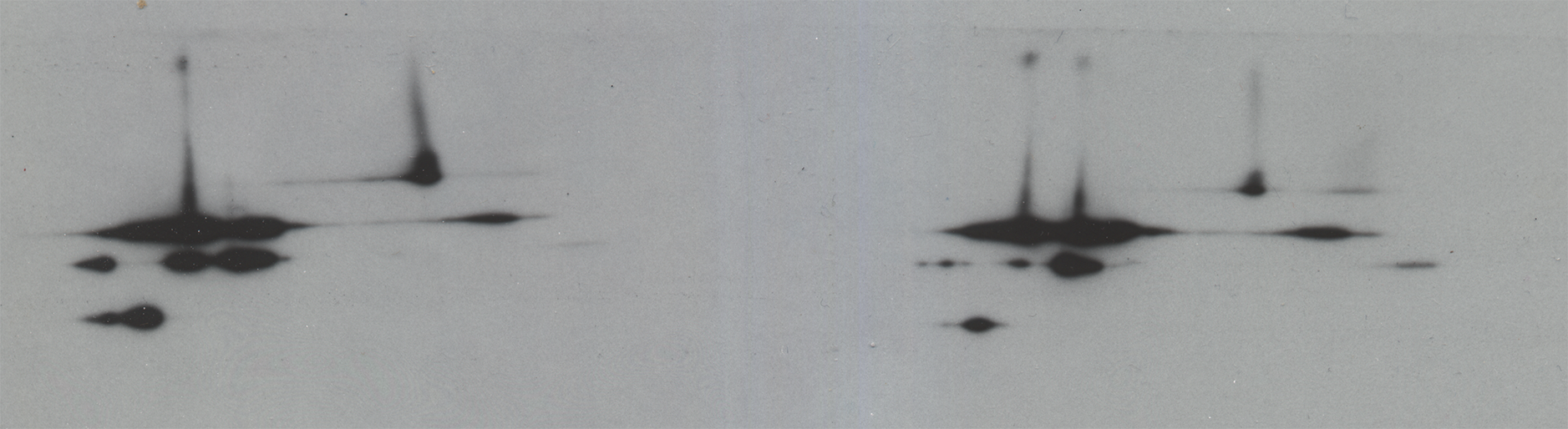

Supplement: Figure 3—source data 2. [file elife-85779-fig3-data2.zip › Figure 3 Source Data Unmarked/Figure 3C Source Data 5.tif]

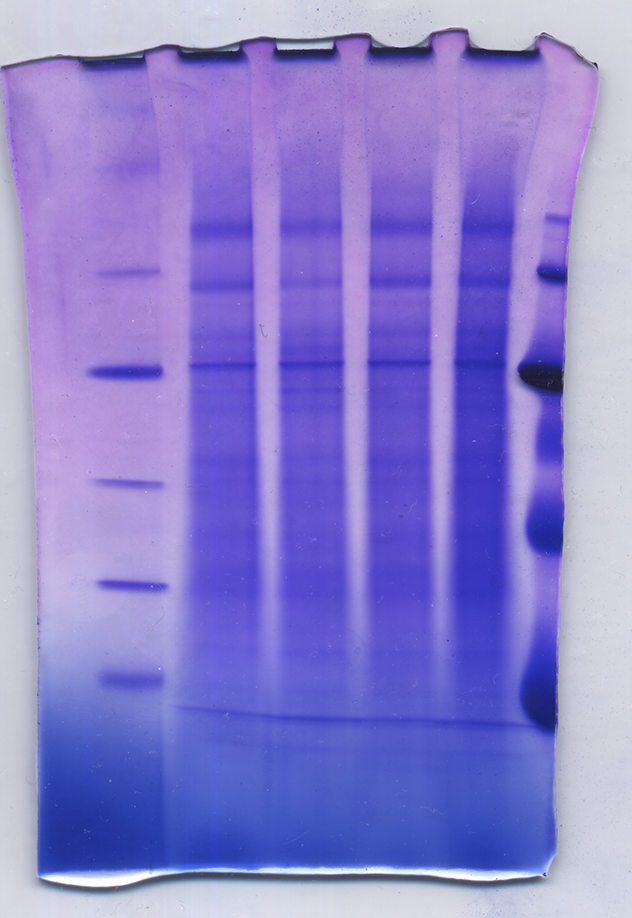

Supplement: Figure 3—source data 2. [file elife-85779-fig3-data2.zip › Figure 3 Source Data Unmarked/Figure 3C Source Data 1.tif]

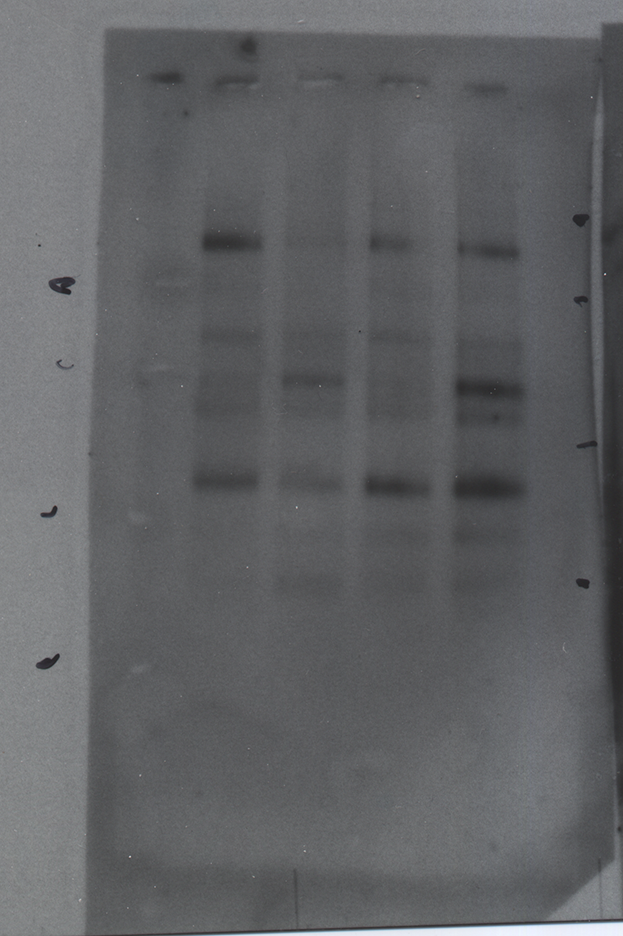

Supplement: Figure 3—source data 2. [file elife-85779-fig3-data2.zip › Figure 3 Source Data Unmarked/Figure 3C Source Data 2.tif]

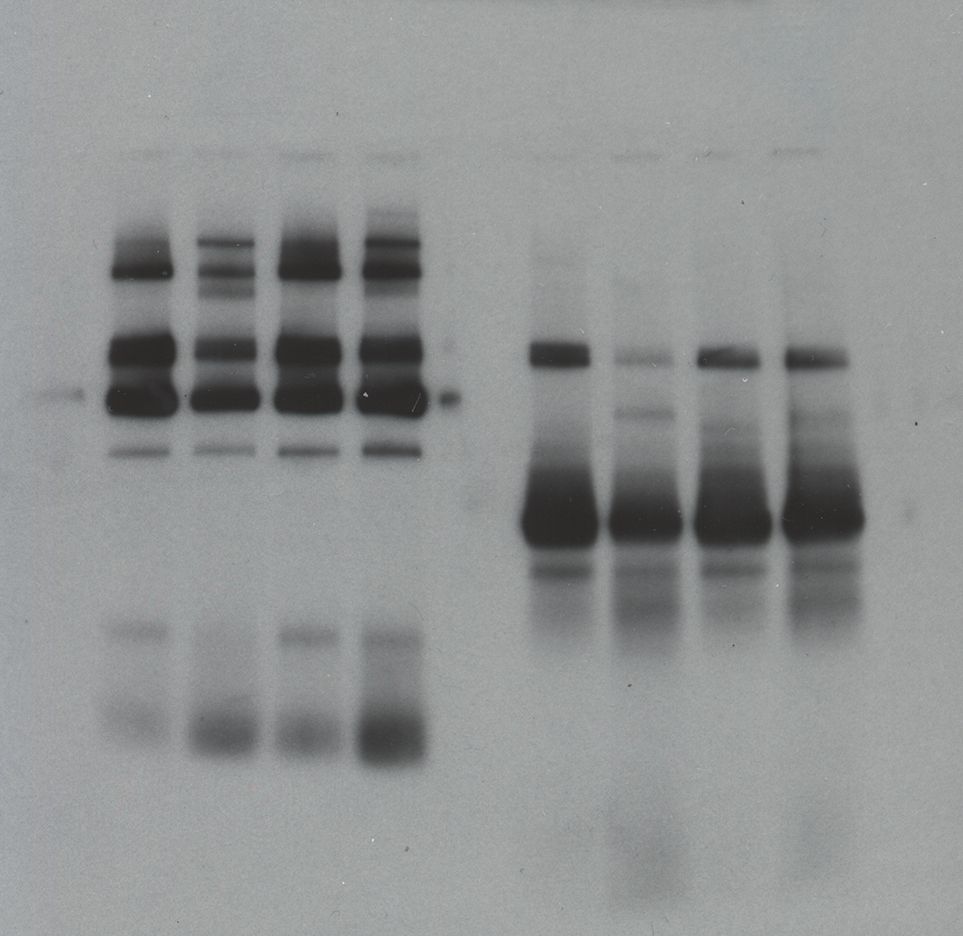

Supplement: Figure 3—source data 2. [file elife-85779-fig3-data2.zip › Figure 3 Source Data Unmarked/Figure 3C Source Data 3.tif]

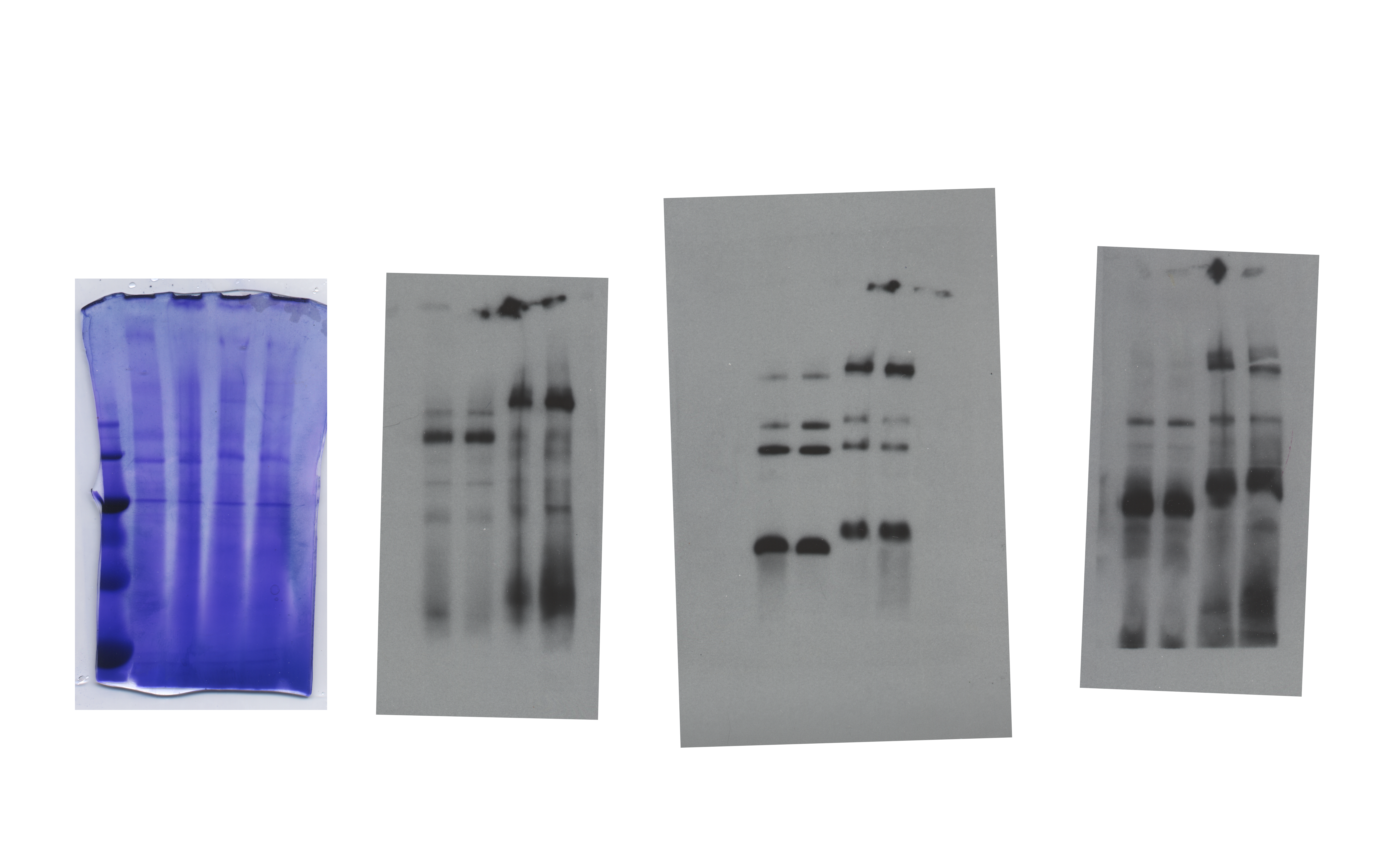

Supplement: Figure 3—figure supplement 1—source data 1. [file elife-85779-fig3-figsupp1-data1.zip › Fig 3 Fig Supple 1 Source Data.tif]

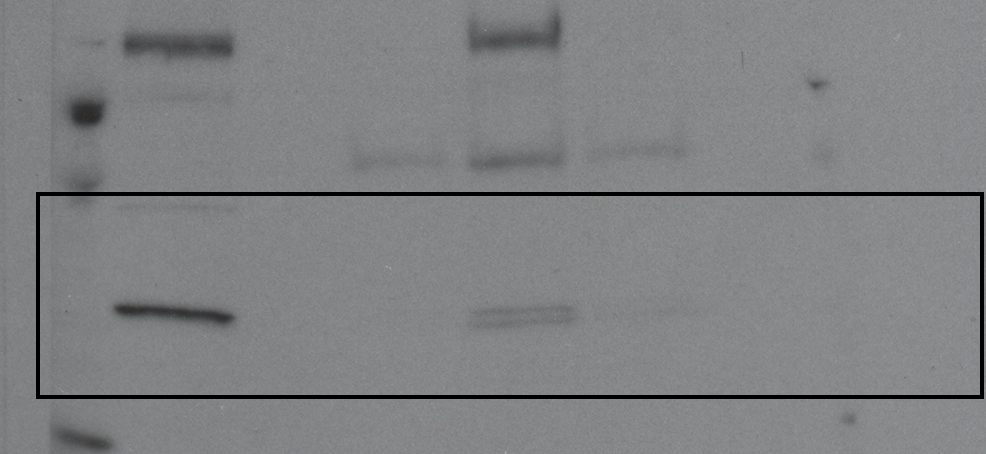

Supplement: Figure 3—figure supplement 3—source data 1. [file elife-85779-fig3-figsupp3-data1.zip › Fig 3 Fig Supple 3 Source Data/Fig 3 Fig Supple 3 Source Data 2.tif]

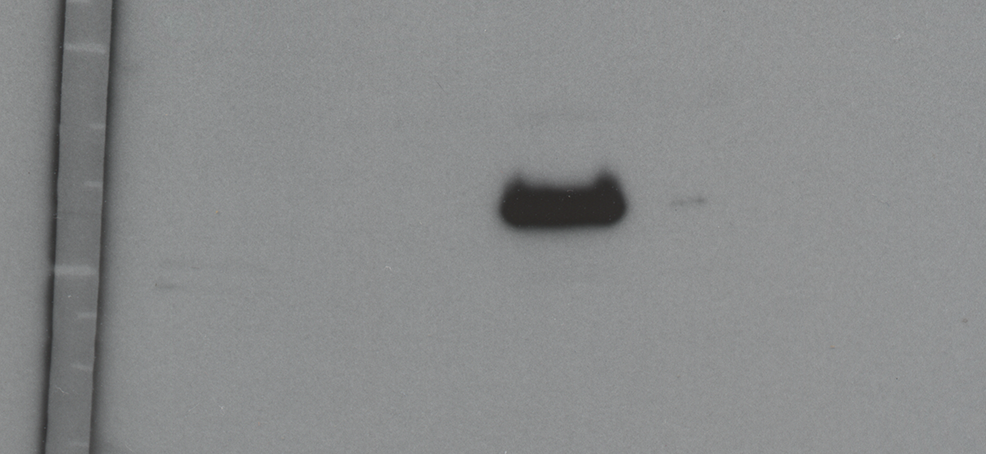

Supplement: Figure 3—figure supplement 3—source data 1. [file elife-85779-fig3-figsupp3-data1.zip › Fig 3 Fig Supple 3 Source Data/Fig 3 Fig Supple 3 Source Data 1.tif]

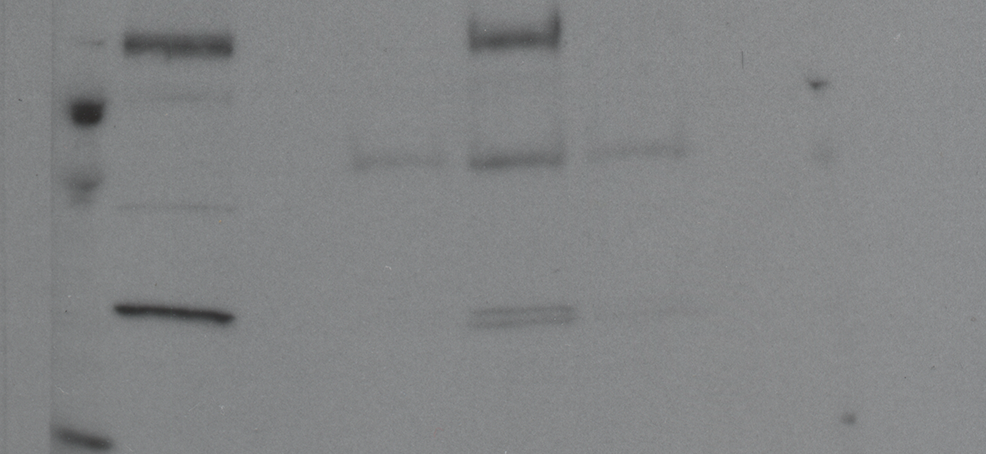

Supplement: Figure 3—figure supplement 3—source data 2. [file elife-85779-fig3-figsupp3-data2.zip › Fig 3 Fig Supple 3 Source Data Unmarked/Fig 3 Fig Supple 3 Source Data 2.tif]

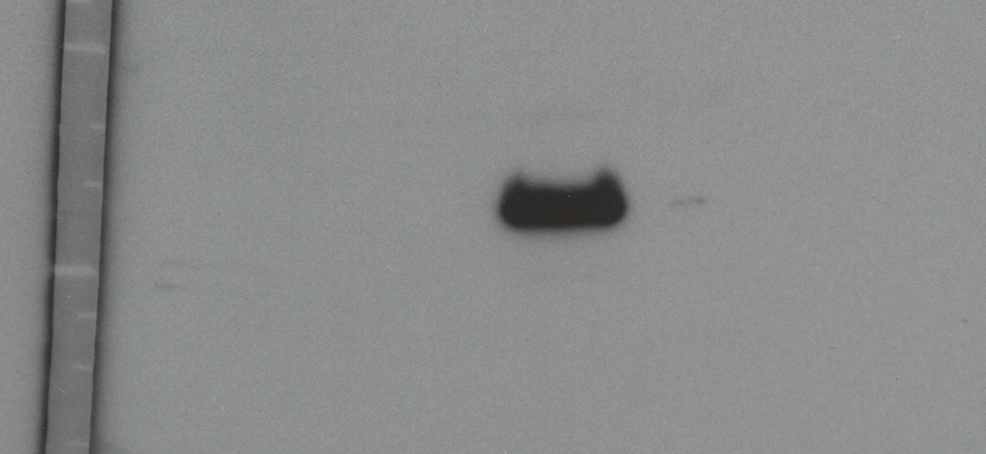

Supplement: Figure 3—figure supplement 3—source data 2. [file elife-85779-fig3-figsupp3-data2.zip › Fig 3 Fig Supple 3 Source Data Unmarked/Fig 3 Fig Supple 3 Source Data 1.tif]

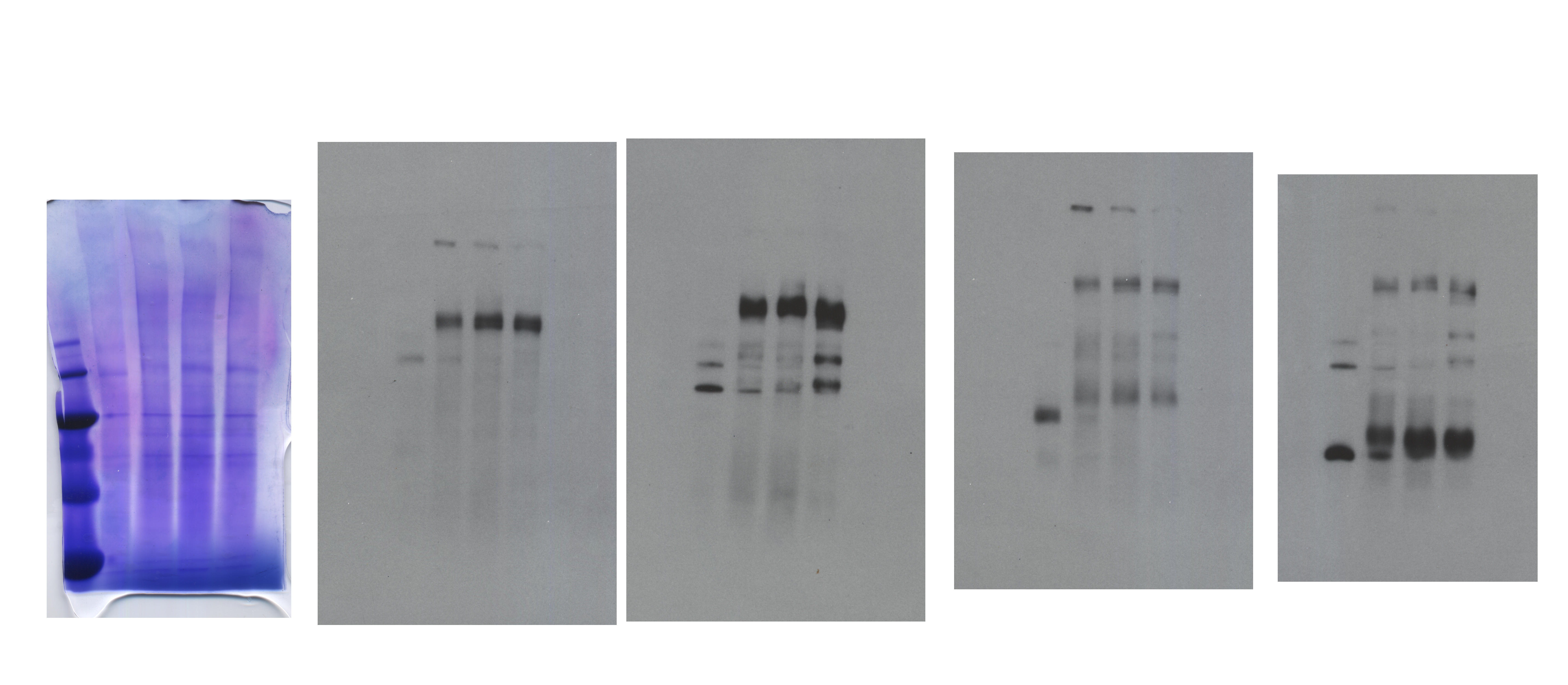

Supplement: Figure 4—source data 1. [file elife-85779-fig4-data1.zip › Figure 4F Source Data 1.tif]

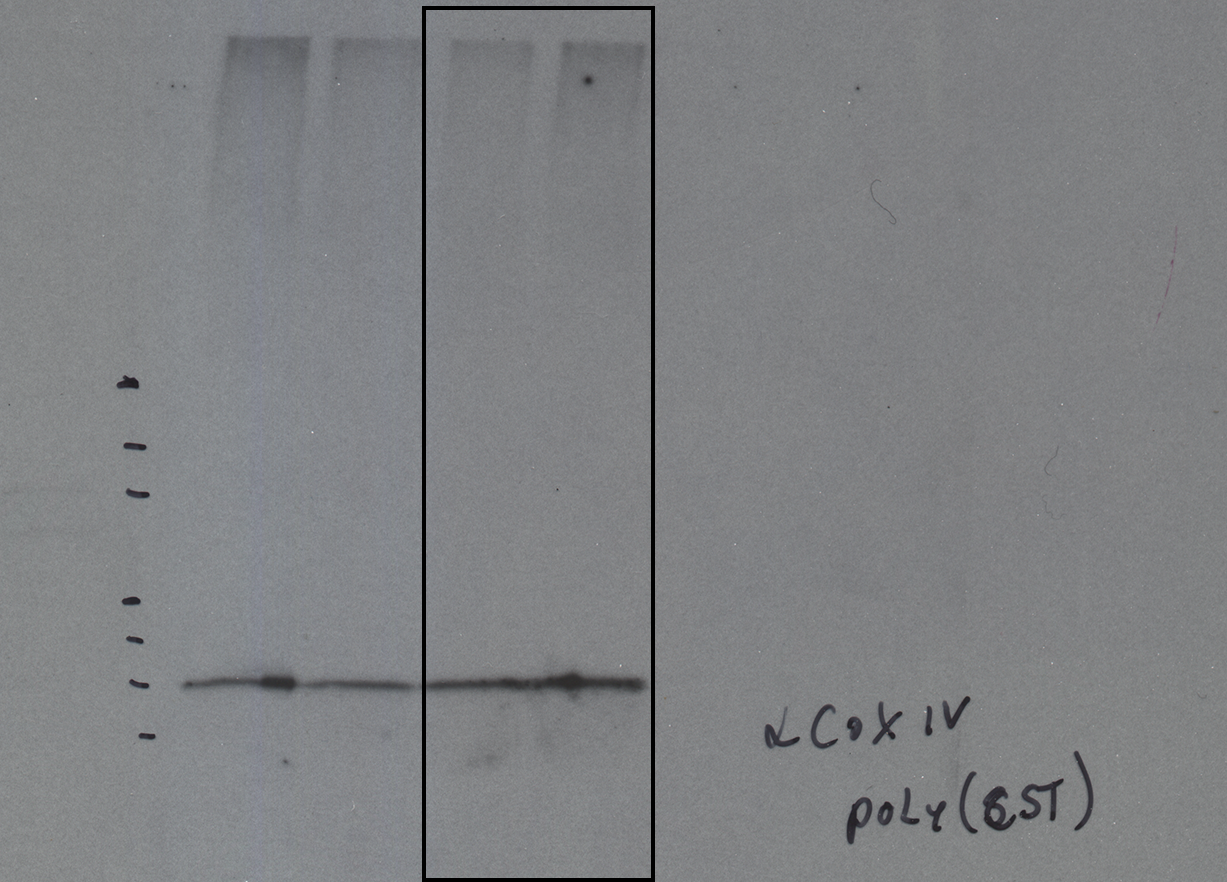

Supplement: Figure 5—figure supplement 2—source data 1. [file elife-85779-fig5-figsupp2-data1.zip › Fig 5 Fig Supple 2 Source Data/Fig 5 Fig Supple 2 3.tif]

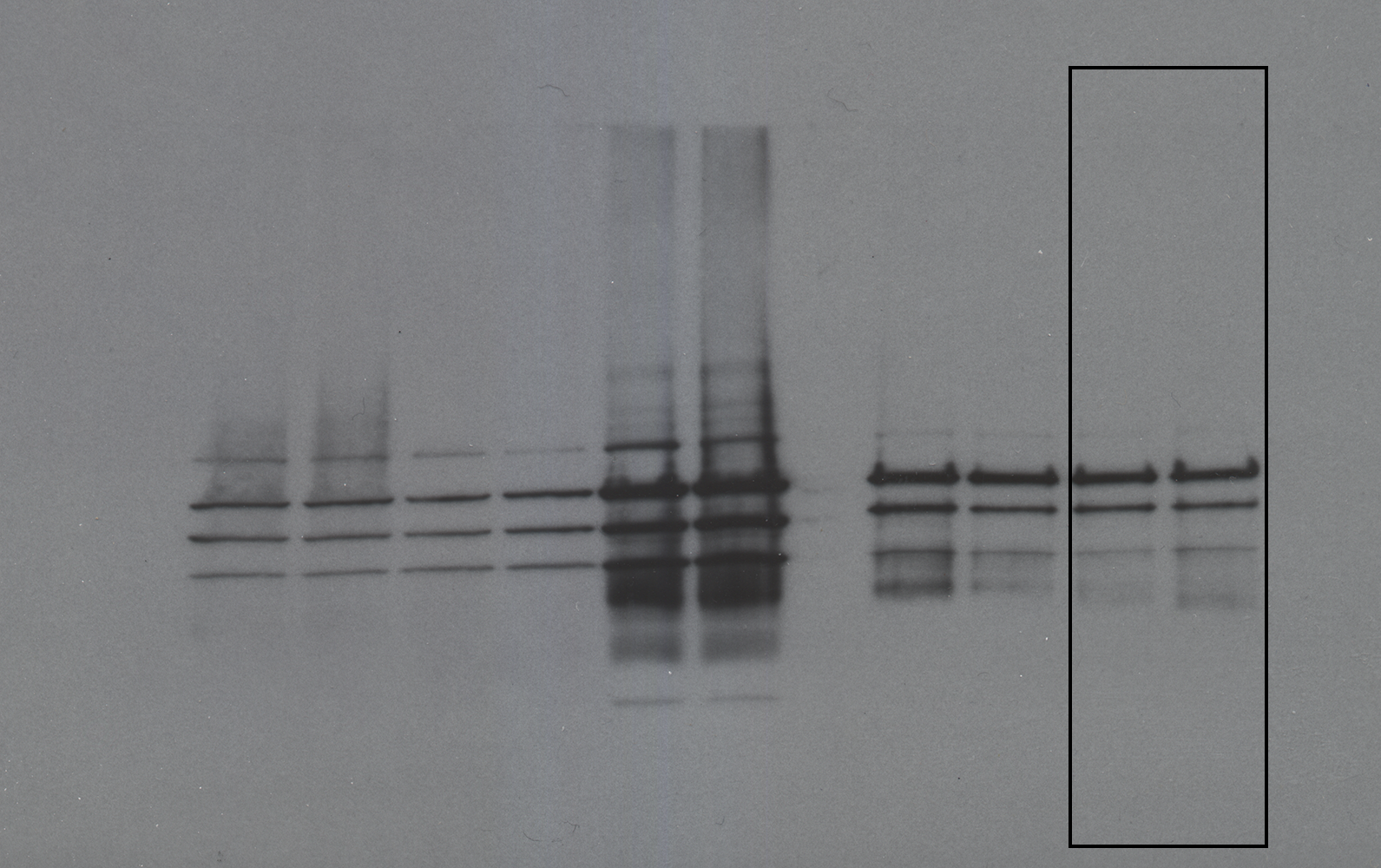

Supplement: Figure 5—figure supplement 2—source data 1. [file elife-85779-fig5-figsupp2-data1.zip › Fig 5 Fig Supple 2 Source Data/Fig 5 Fig Supple 2 2.tif]

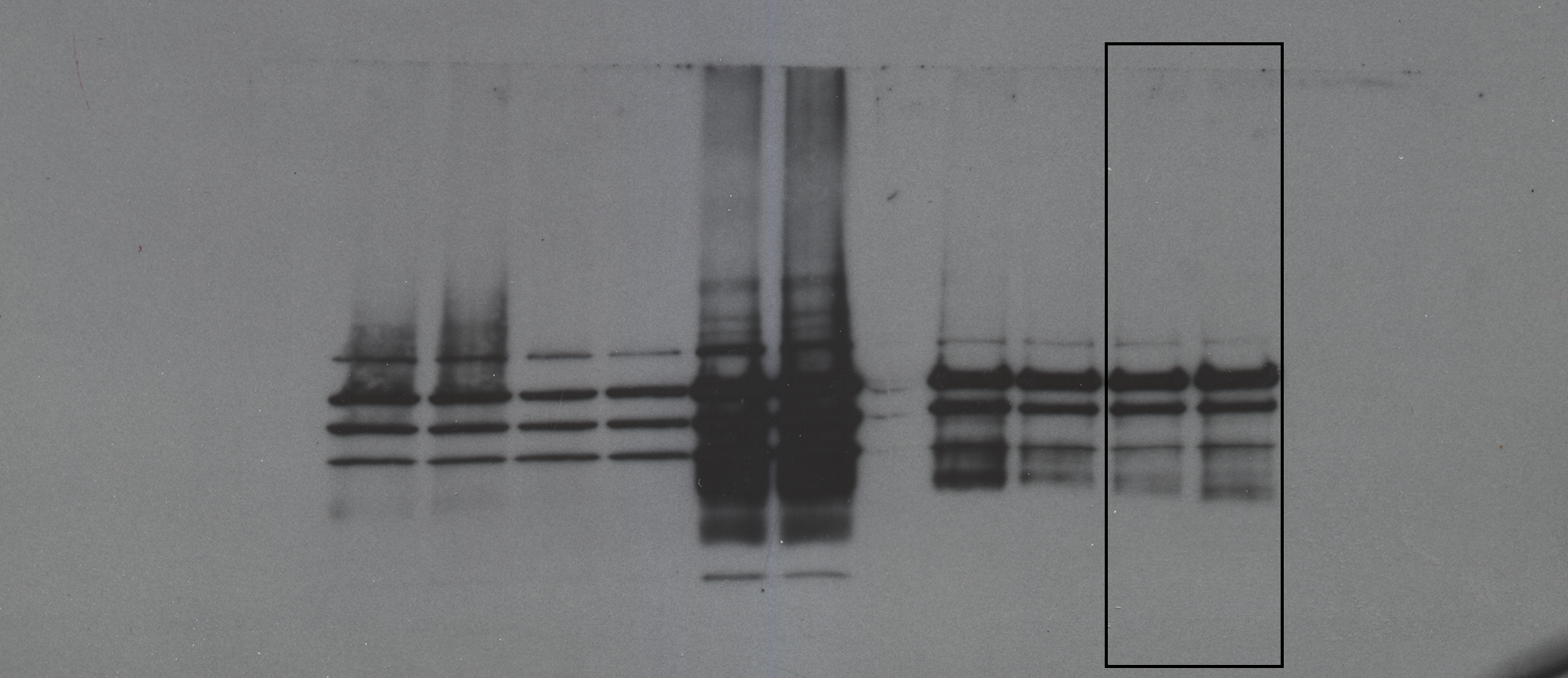

Supplement: Figure 5—figure supplement 2—source data 1. [file elife-85779-fig5-figsupp2-data1.zip › Fig 5 Fig Supple 2 Source Data/Fig 5 Fig Supple 2 1.tif]

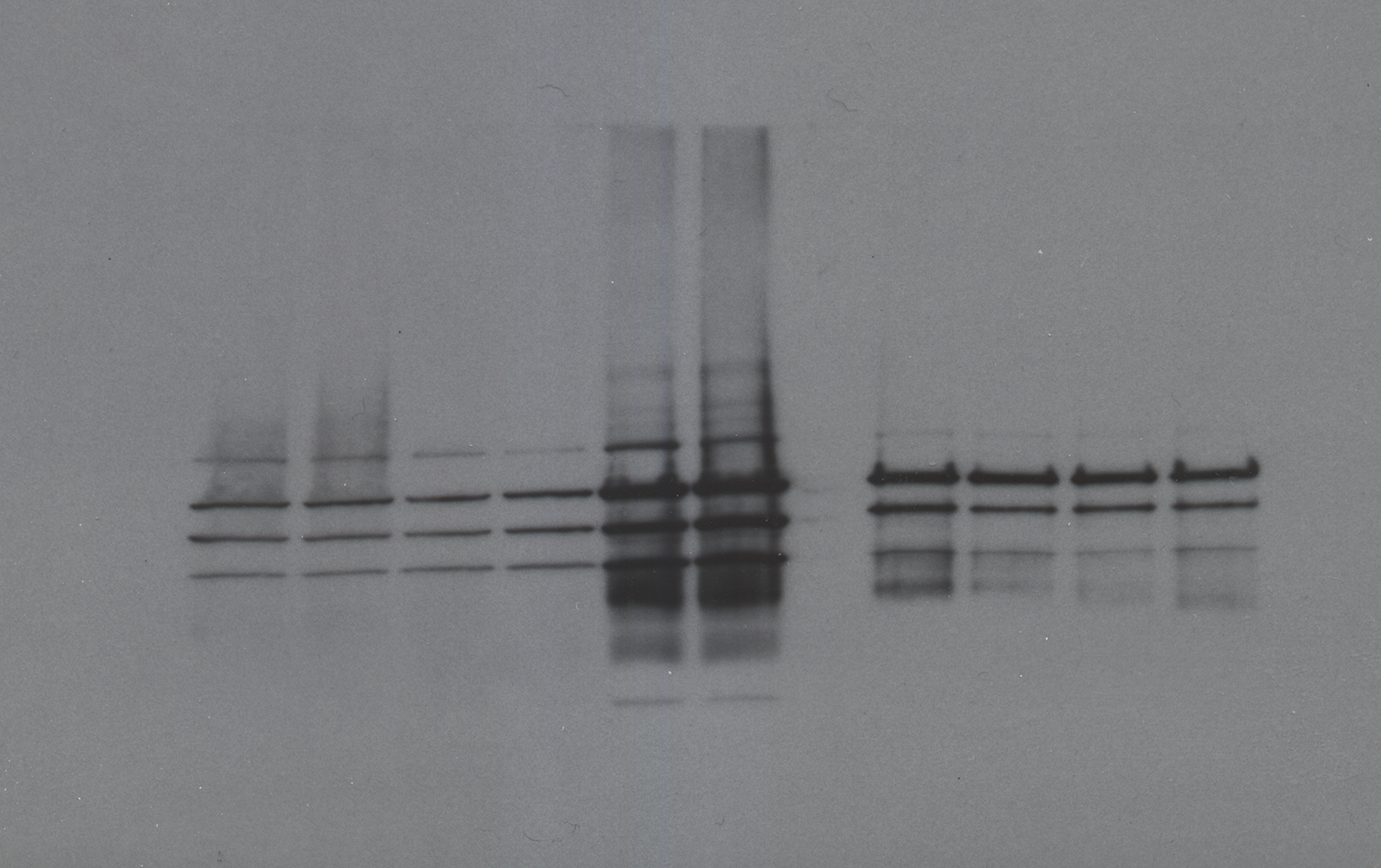

Supplement: Figure 5—figure supplement 2—source data 2. [file elife-85779-fig5-figsupp2-data2.zip › Fig 5 Fig Supple 2 Source Data Unmarked/Fig 5 Fig Supple 2 Source Data 2.tif]

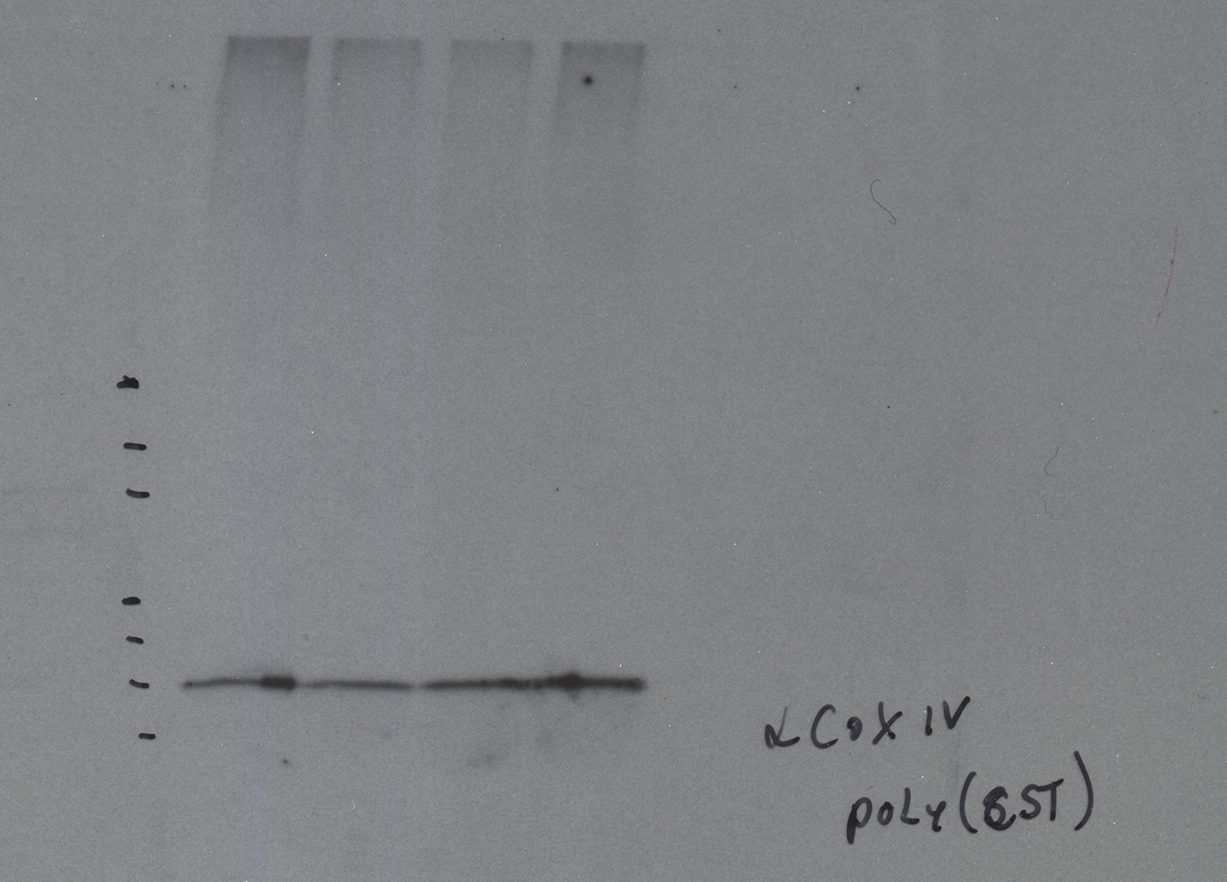

Supplement: Figure 5—figure supplement 2—source data 2. [file elife-85779-fig5-figsupp2-data2.zip › Fig 5 Fig Supple 2 Source Data Unmarked/Fig 5 Fig Supple 2 Source Data 3.tif]

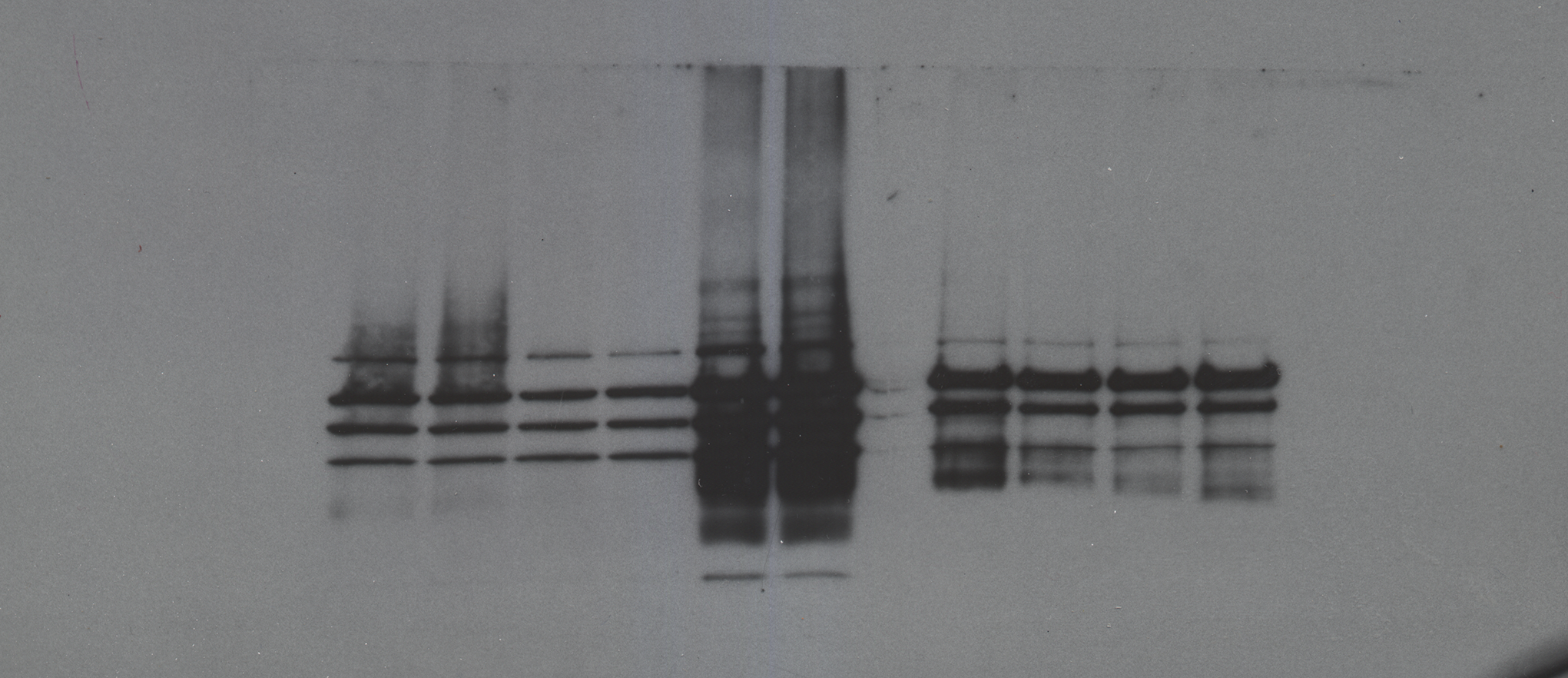

Supplement: Figure 5—figure supplement 2—source data 2. [file elife-85779-fig5-figsupp2-data2.zip › Fig 5 Fig Supple 2 Source Data Unmarked/Fig 5 Fig Supple 2 Source Data 1.tif]

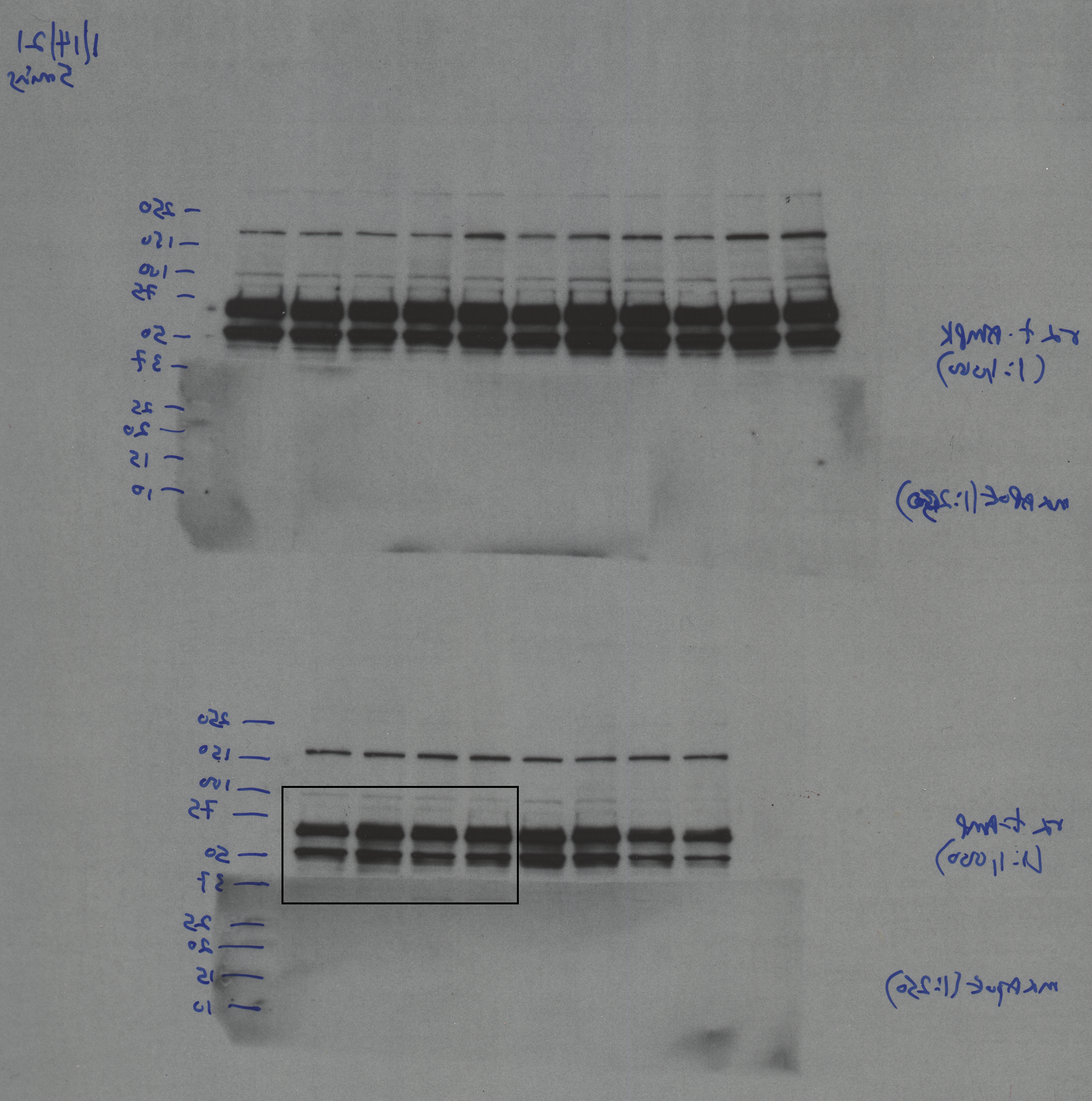

Supplement: Figure 5—figure supplement 5—source data 1. [file elife-85779-fig5-figsupp5-data1.zip › Fig 5 Fig Supple 5 Source Data/Fig 5 Fig Supple 5 Source Data 1.tif]

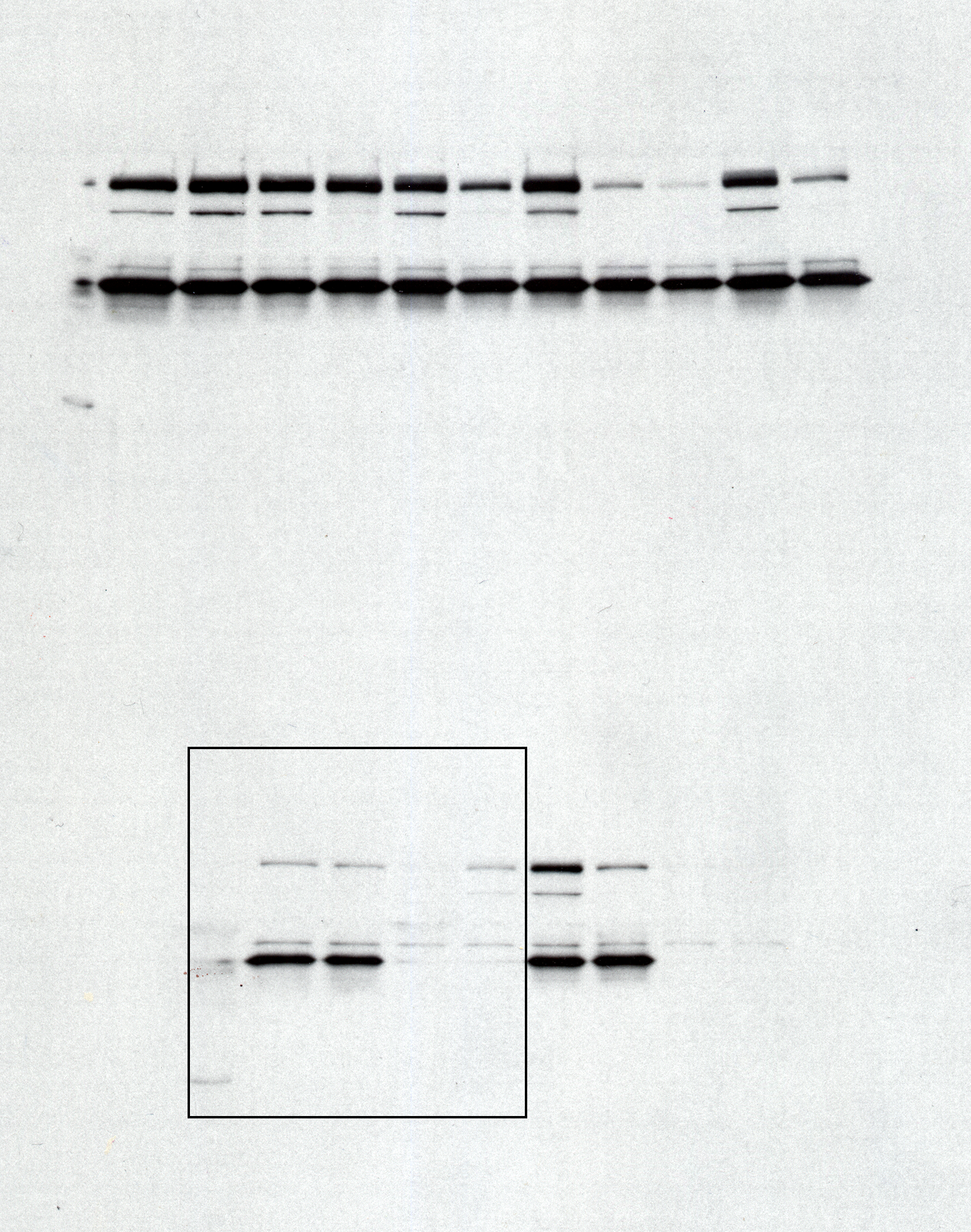

Supplement: Figure 5—figure supplement 5—source data 1. [file elife-85779-fig5-figsupp5-data1.zip › Fig 5 Fig Supple 5 Source Data/Fig 5 Fig Supple 5 Source Data 2.tif]

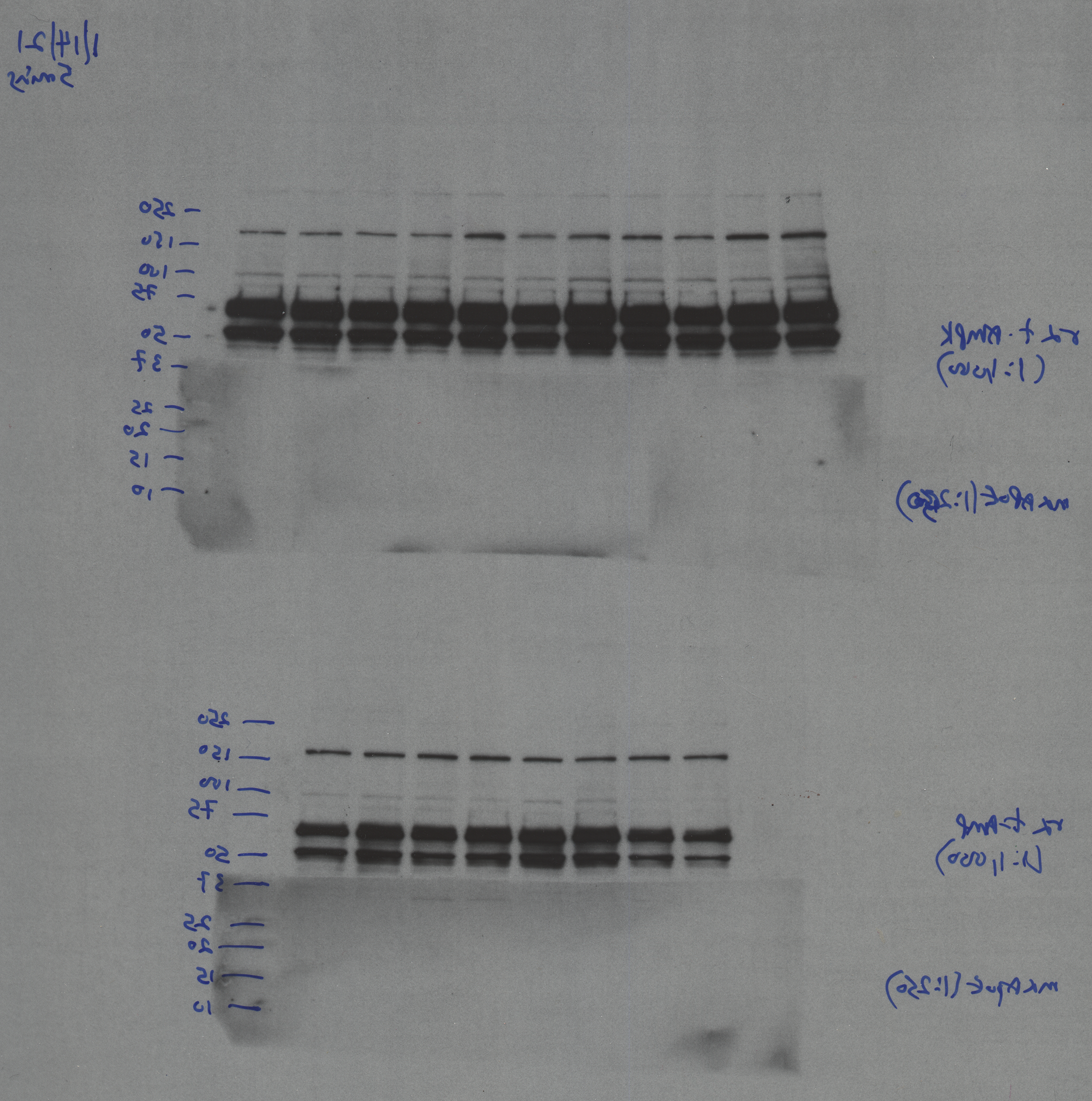

Supplement: Figure 5—figure supplement 5—source data 2. [file elife-85779-fig5-figsupp5-data2.zip › Fig 5 Fig Supple 5 Source Data Unmarked/Fig 5 Fig Supple 5 Source Data 1.tif]

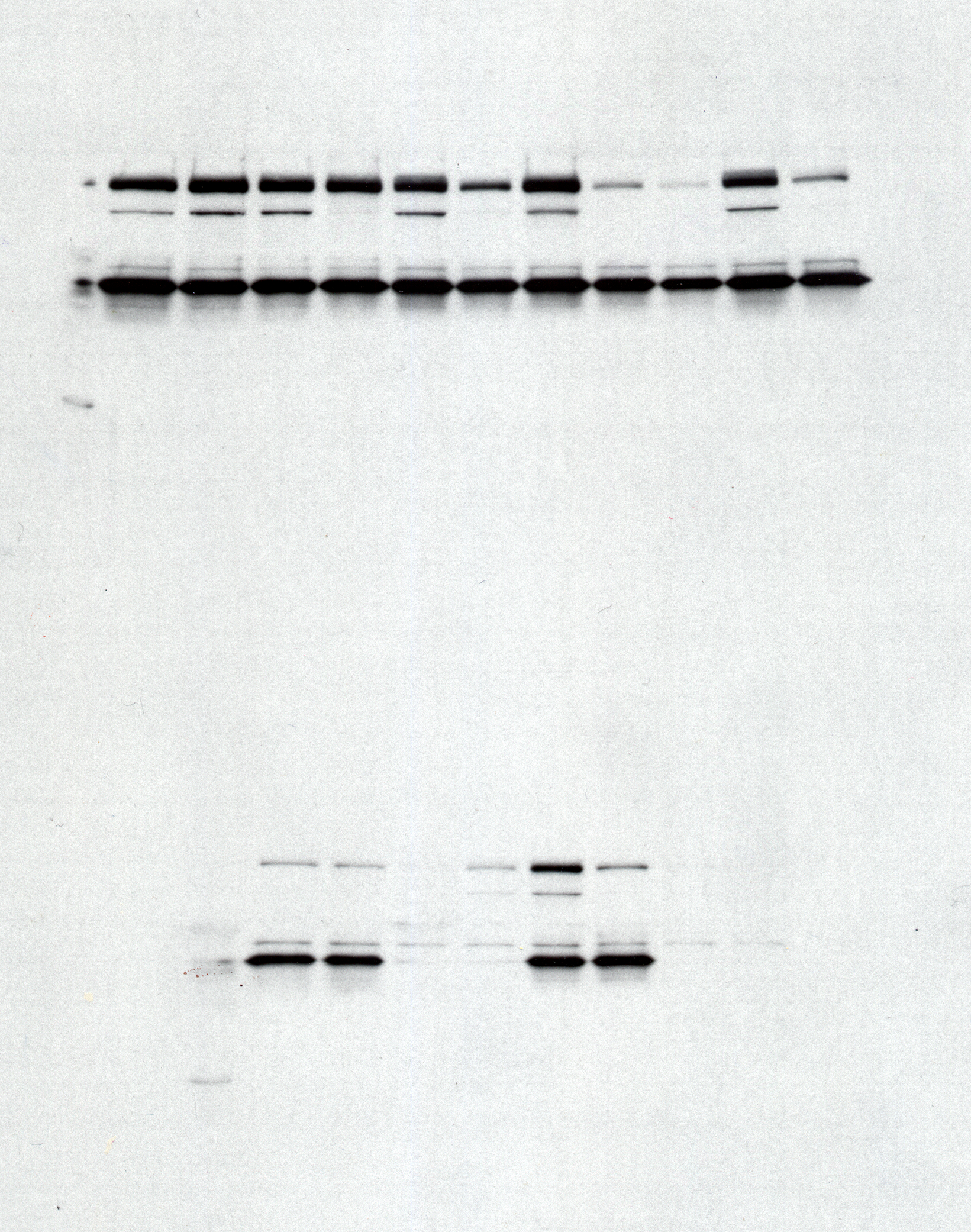

Supplement: Figure 5—figure supplement 5—source data 2. [file elife-85779-fig5-figsupp5-data2.zip › Fig 5 Fig Supple 5 Source Data Unmarked/Fig 5 Fig Supple 5 Source Data 2.tif]
